# Special Issue “*Drosophila*: A Versatile Model in Biology and Medicine—2nd Edition”

**DOI:** 10.3390/ijms27146409

**Published:** 2026-07-19

**Authors:** Maria Grazia Giansanti, Roberto Piergentili

**Affiliations:** Istituto di Biologia e Patologia Molecolari (IBPM) del Consiglio Nazionale delle Ricerche (CNR), at Dipartimento di Biologia e Biotecnologie, Università Sapienza di Roma, Piazzale Aldo Moro 5, 00185 Rome, Italy

## 1. Introduction: The Importance of Model Organisms in Biomedical Research

Model organisms have played a fundamental role in advancing our understanding of biological processes and human diseases. Because direct experimentation on humans is often limited by ethical and practical considerations, scientists rely on organisms that share conserved cellular and genetic mechanisms with humans. Indeed, classical model organisms such as *Escherichia coli* (bacterium), *Saccharomyces cerevisiae* (yeast), *Caenorhabditis elegans* (roundworm), *Danio rerio* (zebrafish), *Rattus norvegicus* (brown rat), *Mus musculus* (mouse), and *Drosophila melanogaster* (fruit fly) have contributed enormously to modern genetics, developmental biology, neurobiology, and medicine. The utility of a model organism depends on several factors, including ease of maintenance, rapid reproduction, genetic tractability, and the degree of evolutionary conservation with humans. Despite the obvious differences between humans and simpler organisms, many fundamental biological pathways have been remarkably conserved throughout evolution. Therefore, studies in model organisms have frequently provided invaluable insights into human physiology and pathology. Among these systems, *Drosophila melanogaster* has emerged as one of the most powerful experimental platforms for investigating the molecular basis of complex human diseases [1,2,3].

## 2. Advantages of *Drosophila melanogaster* in Biological Research

Since the pioneering work of Thomas Hunt Morgan in the early twentieth century, *Drosophila melanogaster* has become a cornerstone of genetics research. Numerous advantageous characteristics have contributed to making the fruit fly particularly attractive for scientific investigation. Drosophila has a short life cycle of approximately 10 days at 25 °C and produces a large number of offspring, enabling rapid genetic studies. Its laboratory maintenance is cheaper when compared with vertebrate models (e.g., mice), allowing large-scale experiments that would otherwise be prohibitively costly. Moreover, Drosophila researchers can benefit from sophisticated genetic tools including the GAL4/UAS expression system, RNA interference, CRISPR-Cas9 genome editing, and extensive collections of mutant and transgenic lines. These resources facilitate the precise manipulation of gene expression in specific tissues and developmental stages.

Beyond these technical advantages, Drosophila exhibits a surprising degree of biological complexity; it possesses complex organs and physiological systems, including a brain, heart-like dorsal vessel, digestive tract, and innate immune system, which can model important aspects of human biology. The complete sequencing of the Drosophila genome in the year 2000 [4] further contributed to its use in functional genomics and disease modeling. Consequently, Drosophila serves as a bridge between simple cellular models and more complex vertebrate systems, and over the decades it has become a leading organism for studying not only basic biological mechanisms but also the genetic and cellular foundations of human disease [5,6].

The extraordinary value of Drosophila in biomedical research derives largely from the high genetic conservation between flies and humans. Comparative genomic analyses have demonstrated that approximately 75% of human disease-related genes possess recognizable homologs in the Drosophila genome [5]. Although the fly genome contains approximately 14,000 protein-coding genes—considerably fewer than the human genome, ca. 20,000—many essential signaling and metabolic pathways are evolutionarily conserved. Key developmental pathways first identified in Drosophila, including Notch, Hedgehog, Wnt/Wingless, Transforming Growth Factor-β (TGF-β), Janus kinase/Signal Transducer and Activator of Transcription (JAK/STAT), and Hippo signaling, were subsequently identified in regulating critical processes in vertebrates and humans as well. Mutations affecting these pathways are now known to contribute to cancer, developmental disorders, and degenerative diseases. The conservation extends beyond individual genes to entire molecular networks. For example, mechanisms controlling cell proliferation, apoptosis, DNA repair, protein homeostasis, and neuronal communication are highly similar between flies and humans. This evolutionary conservation enables researchers to introduce human disease-associated mutations into flies and investigate their functional consequences in vivo. In many cases, human genes can even replace their fly orthologs, demonstrating the preservation of biological function across hundreds of millions of years of evolution.

Another important advantage is the reduced genetic redundancy of Drosophila. Many human genes belong to large gene families with overlapping functions, complicating genetic analyses. In contrast, the fly often contains a single representative of a gene family, allowing clearer interpretation of experimental results. This simplicity has facilitated the identification of disease mechanisms that would be more difficult to uncover in mammalian systems. On the other hand, it should be noted that this lack of redundancy sometimes hides subtle yet significant differences in the behavior of the gene between vertebrates and invertebrates, a limitation that should always be considered when gene function generalization is hypothesized.

## 3. Modeling Complex Human Diseases in *Drosophila*: Advantages and Limitations

Complex diseases arise from interactions among multiple genes, environmental influences, and cellular pathways. Although Drosophila lacks some human organs and adaptive immune responses, it successfully recapitulates many pathological mechanisms underlying human disease [7]. However, despite the remarkable conservation between fly and human genomes, the use of Drosophila as a disease model is not without limitations. Beyond the lack of redundancy described before, approximately one quarter of human disease genes lack a clear fly orthologue, and many human pathologies involve biological systems that are absent or highly simplified in insects, including the adaptive immune system, lungs, and several endocrine organs [5,8]. Consequently, not all disease mechanisms can be faithfully reproduced in flies. This limitation is particularly relevant for disorders in which tissue-specific physiology, complex immune responses, or human-specific developmental processes play a dominant role. Therefore, findings obtained in Drosophila should not be viewed as direct representations of human pathology but rather as powerful tools for identifying conserved molecular mechanisms that can subsequently be validated in mammalian systems.

Nonetheless, the ability to combine genetic conservation with powerful experimental tools has transformed Drosophila into an essential model for studying multifactorial diseases. Researchers can perform genome-wide genetic screens to identify modifiers of disease phenotypes, uncover novel disease-associated genes, and investigate interactions between genetic and environmental factors. Consequently, the fruit fly has become a versatile platform for modeling neurodegenerative disorders, cancer, metabolic diseases, cardiovascular dysfunction, and infectious diseases [8,9].

In neurodegenerative diseases, transgenic flies expressing human disease-related proteins have provided valuable insights into disease progression [10]. Models of Alzheimer’s disease expressing human amyloid-β peptide or tau protein exhibit neuronal degeneration, locomotor defects, and reduced lifespan [11]; similarly, expression of mutant α-synuclein produces phenotypes resembling Parkinson’s disease, including dopaminergic neuron loss and motor impairment, paralleling their human counterparts in this way. However, the fly brain lacks the structural and cellular complexity of the human cortex. Consequently, while Drosophila is exceptionally useful for identifying conserved molecular pathways, it cannot fully replicate the cognitive and behavioral manifestations of human dementia. The strength of the model therefore lies in mechanistic discovery rather than direct clinical prediction [10,12].

Cancer research has also benefited substantially from Drosophila studies. Fly models have elucidated mechanisms of tumor initiation, cell competition, metastasis, and interactions between oncogenes and tumor suppressors [13]. The identification of the Hippo pathway in Drosophila represents a landmark example of how fly genetics can reveal fundamental mechanisms controlling tissue growth and cancer development [14]. Nevertheless, translating findings from fly tissues to human tumors remains challenging because cancer progression is strongly influenced by interactions with the tumor microenvironment and immune system, features that are less complex in Drosophila.

Metabolic disorders such as obesity and diabetes can also be investigated in flies. Despite anatomical differences, insulin signaling pathways are strongly conserved. Manipulation of insulin-like peptides and nutrient-sensing pathways produces phenotypes analogous to insulin resistance and metabolic syndrome in humans [15]. Yet, unlike humans, flies do not develop obesity and diabetes through identical physiological mechanisms. Thus, the value of Drosophila lies primarily in identifying conserved signaling networks rather than reproducing every aspect of human metabolic disease.

## 4. Conclusions

The remarkable success of *Drosophila melanogaster* as a model organism stems from its unique combination of experimental accessibility, sophisticated genetic tools, and extensive evolutionary conservation with humans. As genomic technologies continue to advance, Drosophila will remain an indispensable platform for understanding human disease and identifying novel therapeutic strategies. The continued relevance of *Drosophila melanogaster* in biomedical research is occasionally questioned due to emerging technologies such as human-induced pluripotent stem cells (iPSCs), organoids, single-cell omics, and artificial intelligence-driven disease modeling. At first glance, these approaches appear to offer more physiologically relevant alternatives for studying human disease. However, such technologies often lack the speed, scalability, and genetic tractability that characterize the fly model. Rather than competing with these systems, Drosophila increasingly functions as part of an integrated experimental pipeline in which candidate disease genes identified through human genomic studies are rapidly tested in vivo before validation in mammalian models. In the era of precision medicine, one particularly promising application is the generation of personalized disease models. Human genetic variants discovered through sequencing can be introduced into flies, allowing rapid functional assessment of their pathogenicity. This approach has already contributed to the interpretation of rare disease mutations and may become increasingly important as clinical genomics expands. Therefore, the future of Drosophila research is unlikely to depend on its ability to replace mammalian models, but rather on its capacity to complement them by providing rapid mechanistic insights that would otherwise be difficult or costly to obtain.

In this Special Issue, all this is remarkably illustrated, with contributions spanning immune defense, stress tolerance, senescence, chromatin dynamics, heart development, behavior, mitochondrial biology, intestinal homeostasis, neuronal health and function, and metabolic changes due to dietary modification. The contributions are freely available at the link https://www.mdpi.com/journal/ijms/special_issues/7DL50ZC511 (accessed on 7 July 2026), and a comprehensive list is reported below. We are grateful to all authors who submitted their work and supported this collection and to the *International Journal of Molecular Sciences* staff, whose help was invaluable in the success of this editorial project.
